# The paradox of progress: structured football, self-efficacy, and the Dunning-Kruger effect – a randomized controlled trial

**DOI:** 10.3389/fpsyg.2025.1674900

**Published:** 2025-11-13

**Authors:** Tore Aune, Jarl Magnus Knutsen, Bradley Douglass, Paul Harald Pedersen, Pål Lagestad

**Affiliations:** 1 Faculty of Nursing and Health Sciences, Nord Universitetet, Bodø, Norway; 2 Department of Psychology, Norwegian University of Science and Technology, Trondheim, Norway; 3 Coerver Coaching & DLT Systems AS, Trondheim, Norway; 4 Football for Public Health (FFF), Steinkjer, Norway; 5 Physical Education and Sport Science, Faculty of Teacher Education and Arts, Nord University, Levanger, Norway

**Keywords:** children, football, social self-efficacy, perceived competence, emotion regulation

## Abstract

**Introduction:**

Structured sports interventions are widely believed to enhance self-efficacy and emotion regulation in children. However, few randomized controlled trials (RCTs) have rigorously tested these assumptions, and even fewer have explored the psychological mechanisms underlying unexpected or paradoxical outcomes. This study investigated the effects of an 11-session football-based intervention on social self-efficacy, perceived competence, and emotion regulation strategies in children aged 9–12, with the potential influence of the Dunning-Kruger Effect.

**Methods:**

One hundred and five children (68 in the intervention group, 37 in the control group) participated in a 4-month RCT. The intervention group received 11 structured football sessions (75 min each) incorporating cognitive-behavioral skills training. The outcomes related to social self-efficacy, perceived competence, cognitive reappraisal, and expressive suppression were measured at pre- and post-test using validated self-report scales. Mediation analysis examined whether cognitive reappraisal accounted for the intervention effects.

**Results:**

Contrary to the expectations, the control group reported significantly higher social self-efficacy and perceived competence at post-test than the intervention group. Furthermore, cognitive reappraisal did not mediate the relationship between group assignment and self-efficacy. No significant correlations were found between cognitive reappraisal and expressive suppression. We argue that these findings may reflect the Dunning-Kruger effect, whereby intervention participants, having gained new skills and self-awareness, became more critical and accurate in their self-assessments, resulting in lower self-reported efficacy and competence.

**Discussion:**

Considering the results, we conclude that structured football interventions may inadvertently heighten children’s self-awareness of their limitations, leading to more modest self-evaluation of efficacy and competence. These findings underscore the importance of considering meta-cognitive shifts and the Dunning-Kruger effect when interpreting intervention outcomes, suggesting that self-report measures may not fully capture genuine skill gains in youth interventions.

## Introduction

The symbiotic relationship between sports practice and psychological well-being has emerged as a key focus of academic and public attention (Martín-Rodríguez et al., 2024). Competent functioning in academia, sport, and society requires skills and self-belief of efficacy to be used effectively (Chang et al., 2023; Ekelund et al., 2023). Operative efficacy requires continuously improving multiple subskills (e.g., emotion regulation) to manage ever-changing circumstances, which often contain ambiguous, unpredictable, and stressful elements (Aune and Ness, 2025; Bandura, 1986; Prawita and Hariko, 2024). Expectations of personal efficacy determine whether coping behavior will be initiated, how much effort will be expended, and how long it will be sustained in the face of obstacles and aversive experiences (Aune et al., 2020; Bandura, 1977). The objective of this study is to explore the effect of an intervention targeting self-efficacy and emotional regulation among children in a structured football setting.

Self-efficacy, the belief in one’s ability to succeed in specific tasks, is a foundational component of children’s psychosocial development. It influences how children approach challenges, set goals, and persevere through difficulties, shaping their academic, social, skills, and emotional outcomes (Masten, 2001). High self-efficacy is linked to resilience (Masten and Barnes, 2018), enabling children to view setbacks as opportunities for growth rather than overwhelming obstacles. Among its various domains, social self-efficacy - confidence in one’s ability to navigate social interactions - is particularly critical during childhood, as it fosters peer relationships and emotional well-being (Bandura, 1977; Rashid et al., 2024).

Emotional regulation, the skills and strategies needed to influence and/or modify emotional experiences, have substantial implications for numerous emotional and behavioral outcomes in childhood and adolescence (Gullone and Taffe, 2012). The ability to regulate emotions develops progressively throughout childhood, with significant neurobiological maturation shaping different levels of organization at physiological, cognitive, and behavioral levels during the pre-adolescent period (McRae and Gross, 2020). By ages eight or nine, children have typically learned to regulate their emotions through cognitions or thoughts about themselves, their feelings, or others, marking an important shift from external to internal regulation strategies (Thompson, 2014). This developmental progression continues throughout the pre-adolescent period, making ages 9–12 a critical window for intervention efforts that can establish healthy regulatory patterns with potentially lifelong benefits.

Emotion regulation strategies are closely tied to self-efficacy, including cognitive reappraisal (reinterpreting situations to reduce their emotional impact) and expressive suppression (inhibiting emotional expression). Research suggests that effective emotion regulation enhances children’s ability to manage social challenges and maintain positive self-perceptions. For instance, Liu et al. (2017) found that higher levels of social self-efficacy were associated with more adaptive emotion regulation strategies, such as cognitive reappraisal, among adolescents. Moreover, the use of emotion regulation strategies has been shown to have an indirect influence on perceived stress through resilience. Emotional intelligence has been found to share a positive correlation with both reappraisal and resilience (Thomas and Zolkoski, 2020). In contrast, lower social self-efficacy is associated with the increased use of maladaptive strategies, such as expressive suppression, which can negatively impact interpersonal relationships (Bandura, 2018). Chang et al. (2023) demonstrated that adolescents who employ effective emotion regulation strategies tend to experience better social outcomes and possess higher self-esteem, underscoring the critical role of emotion regulation in social and emotional development. Furthermore, the relationship between self-efficacy and emotion regulation appears to be reciprocal; effective emotion regulation enhances individuals’ self-efficacy, while heightened self-efficacy, in turn, provides a protective factor against emotional dysregulation (Bandura, 1986). Collectively, these findings highlight the importance of the dynamic interplay between self-efficacy and emotion regulation in promoting emotional well-being and facilitating positive social interactions (Chang et al., 2023; Bandura, 1986).

Furthermore, social support is pivotal in reinforcing social self-efficacy by providing children with the requisite resources and encouragement to overcome interpersonal difficulties (Aune et al., 2020; Prawita and Hariko, 2024). Assessing a sample of 252 adolescents, Prawita and Hariko (2024) showed that self-efficacy and family social support simultaneously had a significant relationship with students’ psychological well-being. Aune et al. (2020), using a sample of more than 8,000 older children and adolescents, demonstrated how both social support and social self-efficacy among adolescents mitigated the effect of adverse life events on the development of social anxiety. More specifically, when adolescents experience more frequent adverse life events, social support functions as a significant buffer or protective mechanism against social anxiety symptoms. In addition, social self-efficacy emerged as a strong overall protective factor and was also found to have an additive protective effect on social anxiety symptoms. Therefore, the cumulative picture from Aune et al.’s (2020) study demonstrates that, although social support is important, improving social self-efficacy among adolescents has a significantly more potent effect. More importantly, the additive effect of social support and social self-efficacy indicates that these protective internal and distal factors are bilaterally dependent.

Physical activity contexts, particularly structured sports, such as football, offer unique opportunities for developing self-efficacy and emotional regulation. Sports naturally evoke a range of emotional states, from excitement and joy to frustration and disappointment - creating authentic situations for children to practice regulatory skills and to experience coping. The inherent structure of sports training, emphasizing discipline, perseverance, and teamwork, may foster self-regulatory and self-efficacy capabilities that transfer to other domains. When intentionally designed to incorporate psychological skills training, sports-based interventions leverage children’s intrinsic motivation for physical activity while simultaneously addressing psychological development. This integrated approach aligns with the contemporary understanding of self-efficacy and emotional regulation, which involves both physiological and cognitive processes.

Recent evidence highlights the potential benefits and complexities of combining physical activity with psychological therapies for adolescent mental health. Thomas and Zolkoski (2020) systematically reviewed literature integrating physical activity and psychological interventions, including those with adolescent samples. Their analysis found that such combined approaches consistently yielded positive psychological outcomes compared to treatment as usual, irrespective of the specific ‘dose’ of physical activity administered. This suggests that psychosocial mechanisms - particularly self-efficacy and social support - may mediate these effects, rather than the amount of physical activity per se. Consequently, Thomas and Zolkoski (2020) recommend that future studies incorporate both intervention components and robust measures of self-efficacy and social support to better elucidate these pathways.

Applying the PRISMA-ScR guidelines (Tricco et al., 2018), Ekelund et al. (2023) identified 44 studies evaluating promotion, prevention, and treatment interventions for athletes’ mental health. However, only six studies included samples with a mean age below 19, which underscores a notable gap in research targeting younger athletes.

Gabana et al. (2022) tested a six-week gratitude intervention (TAGG) among 18 female high school soccer players (mean age = 16.6), with and without coach participation. The intervention significantly improved state gratitude, mental health, resilience, team cohesion, and coach-athlete relationships, with the most significant benefits observed when the coach was present. Waters et al. (2022) implemented the RISE program - a multicomponent development intervention among 251 male rugby players aged 12–15. The quasi-experimental design revealed a significant reduction in depressive symptoms in the intervention group, but no reduction in anxiety symptoms among high-risk participants. Wong et al. (2022) applied a mindfulness acceptance and commitment (MAC) intervention with seven weekly sessions among five adolescent squash players (male and female, mean age = 15) using a single-case A-B design. The intervention did not produce improvements in perceived stress or experiential avoidance.

Belz et al. (2020) conducted a randomized controlled trial (RCT) with a 90-min group stress-prevention workshop among 97 soccer players (mean age = 15.5). No significant intervention effects were observed on perceived stress, depression, or psychological well-being. Dallmann et al. (2016) evaluated a six-week stress prevention program in a quasi-experimental design with 56 adolescent athletes (mean age = 16.9). No significant effects on perceived stress were identified. Coelho et al. (2012) assessed a cognitive-behavioral intervention among 46 male tennis players (mean age = 17), finding significant improvements in perceived stress and cognitive anxiety for the intervention group compared to controls. Laurin et al. (2008) employed a goal-setting and personal management program with 22 male soccer players (mean age = 17.5), reporting higher positive and lower negative moods in the intervention group compared to the control group.

Applying latent growth modeling in longitudinal tests in a cohort of 79 boys and 108 girls assessed in 5th, 6th, 7th, 9th, and 11th grades, Dishman et al. (2019) showed that physical activity measured objectively by an accelerometer declined most in students who had more significant decreases in self-efficacy, maintained higher perceptions of barriers to physical activity, had more significant declines in enjoyment and fitness goals, and had smaller decreases in appearance and social goals. Employing an RCT field experiment (*n* = 240), Ng-Knight et al. (2022) reported that teachers reported a positive effect of self-regulation in an 11-week introductory course of Taekwondo among children 7–11 years old.

In summary, the current evidence base for mental health interventions in a sports setting remains inconclusive due to several methodological limitations. Many studies rely on small sample sizes and frequently include only male or female participants. Additionally, using rigorous randomized controlled trial (RCT) designs is infrequent (Ekelund et al., 2023), reducing the strength of causal inferences that can be drawn. Among the 44 studies reviewed by Ekelund et al. (2023), none reported including mediators, which is a critical gap given that mediation analysis is fundamental for elucidating the mechanisms underlying intervention effects. Intervention integrity - specifically, the fidelity with which core components are delivered and the competence of interventionists - also remains underreported. Furthermore, there is a notable paucity of research focusing on younger populations and interventions aimed at health promotion. This is particularly important for evaluating foundational psychological and social skills, such as self-efficacy and emotion regulation, which are closely linked and play a vital role in the development of children and adolescents.

Intrinsic motivation in children is strongly associated with the gratification of basic psychological needs, such as autonomy and competence, as outlined by self-determination theory (Ryan and Deci, 2017). Assessing and controlling intrinsic motivation, which drives individuals to engage in activities due to inherent satisfaction and interest, is particularly critical in research involving children, as parental or guardian involvement often influences their participation. Therefore, rigorous assessment of intrinsic motivation is essential in children’s samples to ensure that observed behavioral outcomes reflect the child’s volition rather than external influences. Therefore, the limited attention to the above-mentioned vital constructs in non-clinical samples constrains our understanding of preventive and promotive mental health strategies. These limitations highlight the need for future research to employ larger, more representative samples, utilize rigorous RCT designs with mediation analyses, systematically assess intervention fidelity and competence, and broaden the focus to include younger populations and health-promoting outcomes (Ekelund et al., 2023).

Cognitive-behavioral therapy (CBT) is an evidence-based approach to enhancing emotional regulation that has been successfully adapted for use with children and adolescents (Kendall and Hedtke, 2006; Stiede et al., 2023). CBT techniques help children identify and challenge unhelpful thoughts, recognize emotional states, and develop adaptive coping strategies (Stallard, 2002; Stiede et al., 2023). Even brief, low-intensity CBT interventions have shown promise for addressing emotional and behavioral difficulties in children (Creswell et al., 2021; Roach et al., 2025). Integrating CBT elements into existing activities, such as sports training, represents an innovative approach that may enhance accessibility and engagement while reducing the stigma associated with traditional mental health interventions. Combining physical activity with targeted psychological components may synergistically enhance self-efficacy and emotional regulation skills development.

Self-efficacy and emotional regulation assessments typically employ developmentally appropriate self-report measures in pre-adolescent populations. The Resilience Scale for Adolescents (READ; Hjemdal et al., 2006) assesses social support and social self-efficacy among older children and adolescents. Complementary assessments, such as the Emotion Regulation Questionnaire for Children and Adolescents (ERQ-CA; Gullone and Taffe, 2012), evaluate two key strategies: cognitive reappraisal and expressive suppression. Together with measures of intrinsic motivation, such as enjoyment, values, and perceived competence, these measures provide a comprehensive assessment of children’s protective factors and strategies to develop self-efficacy and manage emotions and resources supporting their regulatory efforts. Moreover, emotion regulation may facilitate or mediate the development of self-efficacy.

Randomized controlled trials (RCTs) are particularly valuable, as they permit causal inferences about intervention effects while controlling for confounding variables. However, previous research (Burson et al., 2006; Miller et al., 2021) has shown that evidence-based interventions aimed explicitly toward children targeting psychological competencies, such as emotional and self-awareness, can produce the Dunning-Kruger effect (Kruger and Dunning, 1999), in which individuals with increased competence become more aware of their limitations. Specifically, children in an intervention group may have developed a more critical self-assessment of their social self-efficacy, leading to lower self-reported scores despite actual improvements in their skills.

Previous research indicates that interventions that modify self-efficacy may have a positive impact on mental health strategies among adolescents (Aune et al., 2020). Furthermore, in a five-month football intervention, Seabra et al. (2014) found a significant increase in perceived psychological status in the intervention group compared with the control group, as measured by self-esteem and perceived physical competence. Despite the established links between self-efficacy, emotion regulation, and social support, interventions targeting these constructs among children remain underexplored. This study addresses this gap through an RCT examining the effects of a structured football-based intervention program on psychosocial outcomes among children aged 9–12. The program aims to enhance *social self-efficacy* (primary outcome), *perceived competence*, and emotion regulation strategies (*cognitive reappraisal* and *expressive suppression*), while accounting for covariates, such as age, sex, and social support.

First, we hypothesize that the intervention group will report significantly higher levels of social self-efficacy than the control group. Second, we hypothesize that cognitive reappraisal mediates the path from group (independent variable) to social self-efficacy (dependent variable).

## Materials and methods

### Design

The data were derived from a comprehensive experimental intervention study involving a tuition-free football school (Pedersen et al., 2024). The questionnaire had ID numbers linked to the respondents’ names in a password-encrypted data file on OneDrive. The study consisted of an intervention and a control group, utilizing questionnaires and skill tests. The pre- and post-test data collected included information from the Emotion Regulation Questionnaire (ERQ-CA; Gullone and Taffe, 2012) and three of the six IMI subscales - interest/enjoyment, perceived competence, and value/usefulness (McAuley et al., 1989). At post-test, two subscales (Social Support and Social Self-efficacy) from the Resilience Scale for Children and Adolescents (READ: Hjemdal et al., 2006) were included in the questionnaire. The data at the pre-test (Time I) were collected at the football school on November 5th, 2023, while the post-test (Time II) was collected at the football school on February 10th, 2024 (Pedersen et al., 2024; Sørensen et al., 2024). The children were given the questionnaire upon arrival (boys at 12:00 p.m. and girls at 4:00 p.m.). When necessary, the children’s parents assisted their children during this process.

### Participants

Participants were children aged 9–12 who had applied to join a football school free-of-charge. A total of 175 children applied, and 147 (mean age = 10.3, SD = 1.2) were accepted (boys *n* = 101, *M* = 10.3, SD = 1.2; girls *n* = 46, M = 10.5, SD = 1.3) into the school and completed the questionnaire. The number of boys and girls by age is as follows: age 9 boys (*n* = 38), age 9 girls (*n* = 16); age 10 boys (*n* = 26), age 10 girls (*n* = 7); age 11 boys (*n* = 14), age 11 girls (*n* = 6); age 12 boys (*n* = 24), age 12 girls (*n* = 16). One hundred children were randomly assigned to an intervention group, while the other 75 children constituted the control group. With such a strategy, a representative and random sample was included in both groups according to age and gender. This is supported by the fact that independent t-tests showed that there were no significant differences between the intervention group and the control group according to both self-reported activity level at both pre-test (*t* = −0.4, *p* = 0.697) and post-test (*t* = −0.5, *p* = 0.627), but also according to gender (*t* = −0.8, *p* = 0.429) and age (*t* = −0.7, *p* = 0.690) there were no significant differences between the intervention group and the control group. Using power calculations (Cohen, 1988) with the numbers from an earlier intervention study with expected differences between groups (1 = 0.39, *α* = 0.05, *β* = 0.8) and standard deviation (SD = 0.23), we had to have 53 participants to fulfill the criteria for observed power. The control group received no structured sports or psychosocial activities between pre- and posttest, and they did not participate in attention-matched sessions. One hundred and four participants (*n* = 104) attended both the pre-test (Time I) and the post-test (Time II), comprising 36 (34.6%) girls and 68 (65.4%) boys.

### The public health intervention manual (FFF)

The Football for Public Health (FFF) intervention is a structured, football-based program designed to enhance psychosocial skills in children aged 9–12, which was offered to the intervention group. This intervention incorporates seven key components: (1) friendship-building; (2) emotion recognition; (3) social skills; (4) thoughts-feelings and behavior; (5) attention focusing; (6) diaphragmatic breathing; and (7) mindfulness. These components were conducted weekly within 75-min football training sessions, each intervention lasting 6–10 min and facilitated by experienced coaches. The program emphasizes experiential learning through role-playing, breathing exercises, and supplementary homework assignments. Each session was themed, beginning with a 2 to 3-min breathing exercise starting from Session 4. An integral part of the program included a large poster displaying faces that exhibited four emotions: anger; sadness; fear; and happiness. Participants placed a sticker on their most intense emotion before each intervention, reinforcing emotional awareness. Grounded in social learning and cognitive-behavioral theories, the intervention aims to enhance self-efficacy and emotional regulation. Adhering to a manualized format ensures fidelity, focusing on skill acquisition through active engagement and reflection.

### Facilitator training and implementation

Coaches participated in a structured training program consisting of two 2-h lectures focused on the FFF manual. The training included live demonstrations, role-playing exercises, and *in vivo* practice to ensure a high level of procedural fidelity. A designated coach, who held a master’s degree in psychological science, facilitated all psychosocial sessions for the intervention group. Each session lasted 6–10 min and focused on weekly themes from the FFF curriculum. Prior to each weekly session, the coach received a 30-min individualized supervision meeting with the first author—a clinical child psychologist and certified CBT supervisor—conducted either on the same day or the day preceding the session. The supervision sessions addressed the specific weekly theme and provided targeted feedback. Following each intervention session, the first author also delivered feedback directly in person or by phone. The intervention was implemented with two age-based groups who participated in 75-min weekly sessions. During public health activities, participants were further divided into smaller subgroups to facilitate active engagement and peer interaction.

### Adherence, competence, and integrity

Assessment of facilitator adherence and competence is crucial for evaluating the integrity of the intervention and determining whether observed effects stem from the intervention itself or from variations in its delivery. Intervention integrity is defined as the degree to which an intervention is implemented as originally intended (Perepletchkova et al., 2007). Adherence refers to the extent to which the core components are delivered according to protocol, while competence captures the facilitator’s skill and proficiency in executing the intervention (Dusenbury et al., 2003). During the initial training, all coaches—including the primary facilitator—engaged in peer evaluation exercises, assessing both adherence and competence under the supervision of the first author. Coaches used a standardized workbook detailing each session’s curriculum, supplemented by Likert scales (1–5) to rate adherence and competence (see Table 1). Two trained coaches independently rated each intervention session led by the designated facilitator, and the two scores were averaged to yield session-specific summary ratings. If the independent ratings differed by more than one scale point, the discrepancy prompted a joint review with the first author to reach consensus. Although participant engagement and motivation were not directly measured, attendance records indicated that over XXX participants attended more than XXXX training sessions, suggesting consistent involvement across the intervention period.

**Table 1:** Example of session evaluations at FFF.

Session Num.	Skill area	Content example	Quality (5 = best) Scale 1–5	Comments
2	Emotions	Describe situations where you felt angry, happy, scared, sad	1-2 -3 - 4 -5 (A) 1-2 -3 - 4 -5 (C)	
	Emotions	Recognize emotions in others	1-2 -3 - 4 -5 (A) 1-2 -3 - 4 -5 (C)	
	Emotions	What triggers different emotions?	1-2 -3 - 4 -5 (A) 1-2 -3 - 4 -5 (C)	
	Emotions	Place current emotions on board (image)	1-2 -3 - 4 -5 (A) 1-2 -3 - 4 -5 (C)	
Home-Work	Emotions	Practice recognizing emotions in self/others		

A = Adherence; C = Competence.

#### Assessment ratings

In the present study, intervention fidelity was evaluated using two dimensions: adherence (extent to which the planned content was delivered) and competence (quality of instructional delivery). Adherence ratings ranged from 1 (not implemented) to 5 (fully implemented), while competence ratings ranged from 1 (poor instruction) to 5 (excellent instruction). For competence, a score of 1 reflected little or no explanation, demonstrations, examples, or exercises, with minimal or no participant engagement. A score of 2 indicated that some explanations and simple demonstrations or exercises were provided, but with limited participant response. A score of 3 reflected provision of explanations, demonstrations, examples, and exercises, with active participation and some independent practice. A score of 4 indicated clear, effective explanations, easily understandable demonstrations and examples, and active participation by most participants. A score of 5 denoted high-quality instruction, highly informative demonstrations and examples, active participation by most participants, and strong engagement.

For adherence, a score of 1 indicated that the session was not implemented or only minimally implemented. A score of 2 reflected delivery of parts of the planned session or program, whereas a score of 3 indicated that approximately 50% of the session was implemented. A score of 4 corresponded to delivery of most session content (approximately 75%), and a score of 5 indicated that the entire or nearly the entire session or program was implemented. Across all sessions, mean adherence and competence ratings were 4.4 and 4.6, respectively, indicating very good to excellent fidelity in both domains.

### Instruments for measurements

*The Resilience Scale for Children and Adolescents* (READ; Hjemdal et al., 2006). The original READ addresses five factors; the two factors included in this study are titled social self-efficacy (S-SE) (e.g., “I easily make others feel comfortable around me”) and social support family (SSF) (e.g., “In my family, we support each other”). The five response options for each range from “I totally disagree” (1) to “I totally agree” (5). READ shows adequate psychometric properties and high validity compared with measures of mental difficulties (Askeland and Reedtz, 2015; Hjemdal et al., 2006) (see Aune et al. (2020) for a more detailed description of these two scales). Low scores indicate better social self-efficacy and stronger social support. In this study, Cronbach’s alphas for the social self-efficacy and the social support scales were 0.74 and 0.78, respectively.

*The Emotion Regulation Questionnaire for Children and Adolescents* (ERQ-CA; Gullone and Taffe, 2012) is a self-report instrument that measures children’s emotion regulation strategies. The ERQ-CA consists of 10 items assessed on a five-point Likert scale, with responses ranging from 1 (strongly disagree) to 5 (strongly agree). The scale assesses two dimensions of emotion regulation: cognitive reappraisals and expressive suppression (Gullone and Taffe, 2012). The ERQ-CA has demonstrated limited but adequate convergent and discriminant validity in research with children and adolescents (Liu et al., 2017; Martín-Albo et al., 2018; Teixeira et al., 2015). The scale is positively correlated with measures of emotion regulation and negatively correlated with measures of anxiety and depression (Gullone and Taffe, 2012). Examination of the psychometric properties of the Norwegian version of the ERQ-CA (Aune and Ness, 2025) reported a robust two-factor structure model. Test–retest reliability in four months was *r* = 0.50 for CR and *r* = 0.32 for ES, and the ERQ-CA exhibited convergent and concurrent validity with established measures of resilience and motivation. Measurement invariance across sex at the pre-test and intervention groups at the pre-and post-test indicated stability across responses to the ERQ-CA (Aune and Ness, 2025). For this sample, the Cronbach’s alphas for cognitive reappraisals and expressive suppression were 0.75 and 0.76, respectively. Higher scores indicate the use of more cognitive reappraisal and expressive suppression.

*The IMI scale* (McAuley et al., 1989). This self-report multidimensional measurement device is intended to assess participants’ subjective experience related to a target activity in laboratory experiments. It has been used in several experiments on intrinsic motivation and self-regulation (e.g., Cocca et al., 2022; Deci et al., 1994; Ryan and Deci, 2017). The instrument assesses participants’: (1) interest/enjoyment; (2) perceived competence; (3) value/usefulness; (4) effort/importance; (5) pressure/tension; and (6) perceived choice. This study only assesses the first three areas. The interest/enjoyment subscale is a self-report measure of intrinsic motivation. The perceived competence concepts are theorized to be positive predictors of both self-report and behavioral measures of intrinsic motivation. The value/usefulness subscale is used in internalization studies (e.g., Deci et al., 1994), the idea being that people internalize and become self-regulating concerning activities that they experience as functional or valuable for themselves.

The IMI consists of varied items from these subscales, all analytically coherent and stable across various tasks, conditions, and settings (Cocca et al., 2022; Ryan and Deci, 2017; Deci et al., 1994). The questionnaire applied in this study consists of 20 items assessed on a seven-point Likert scale, with responses ranging from 1 (not at all true) to 7 (very true). Interest/enjoyment included seven questions (e.g., “I enjoyed doing this activity very much”). Perceived competence was assessed using six questions (e.g., “I think I am pretty good at this activity”). Value/usefulness included seven questions (e.g., “I believe this activity could be of some value to me”). The reliability analyses revealed Cronbach’s alphas (CAs) of 0.51 for the interest/enjoyment subscale, 0.83 for the perceived competence subscale, and 0.79 for the value/usefulness subscale.

### Procedures and data collection

A pilot study among 17 participants (15 boys and two girls, age 9–12 (SD = 10.3)) from two football teams in a medium-sized city in Norway examined the participants’ understanding of the 20 questions related to IMI and 10 questions related to ERQ-CA. The questionnaire was completed in 4 to 15 min (SD = 11 min). Seven participants noted that some of the 10 ERQ-CA questions were difficult to understand. Specifically, questions 5 and 7–10 were mentioned as challenging. Some parents who observed their children completing the questionnaire also pointed out that these questions were difficult for children aged 9–12 to grasp. However, the questions were not rewritten during this validation phase, allowing the findings to be compared with other studies using the ERQ-CA. The data were collected at the football school on November 5th (Time I) 2023 and on February 10^th^ (Time II) (Pedersen et al., 2024). The children were given the questionnaire upon arrival. When necessary, the children’s parents assisted their children. Especially the youngest children received some support from their guardians during the completion process, and all children were allowed to ask the test administrators questions.

### Statistical analyses

In this study, we chose *not* to replace missing data with mean values or other imputation techniques. This decision was based on the concern that such methods could introduce bias, especially in cases where post-test data were missing. Since we have no empirical basis for estimating what these participants would have answered, inputting their responses could distort the findings. Instead, we applied a *listwise deletion* approach, excluding participants with missing data from the relevant analyses. We considered this to be the most conservative and transparent strategy, given the nature of our data and study design. A chi-square test of independence was conducted to examine the relationship between sex and group, and between age and group. A binary product–moment correlation analysis was performed across all included variables. The Kolmogorov–Smirnov test and the Levene’s test (O’Donoghue, 2012) showed that the assumption of normality and similar variances was not related to the included variables (*p* < 0.05). However, independent t-tests have been shown to produce valid results even when the sample is not normally distributed or with variability in the sample (Lumley et al., 2002; Vincent and Weir, 2012), especially regarding the high number of subjects in the present study. Paired sample *t-*tests were conducted to assess time effects for all dependent variables in both the intervention and control groups. Independent sample t-tests were conducted to check for a statistically significant difference between the intervention and the control groups across the control variables. For control variables, statistically significant differences across the two conditions were included as covariates in the further analyses.

To test the hypothesis that participants in the intervention group will exhibit statistically significantly higher levels of social self-efficacy (the primary outcome variable) compared to those in the control group following the intervention, univariate regression analyses using a general linear model (GLM) were performed, controlling for age, sex, and social support. Separate univariate regression analyses (GLM) were also conducted to test for significant differences in the secondary outcome measures, controlling for age, sex, and other relevant control variables, eventually revealing statistically significant differences between the control and intervention groups. For the primary outcome measure and the secondary outcome measures, the significance levels were set to *p* < 0.05 and *p* < 0.0125, respectively.

The PROCESS macro employs a regression-based path analysis approach. To test for statistical significance and obtain the 95% bias-corrected confidence interval for the indirect effects, standard maximum likelihood bootstrapping was performed by estimating 5,000 bootstrap samples for the hypothesized model using the PROCESS 3.5 macro. The direct effect of *X* quantifies how much two cases that differ by one unit on *X* are estimated to differ on *Y*, independent of all mediators (Hayes, 2018). Bootstrapping was recommended as a resampling method for estimating mediation (Hayes, 2018) If the 95% confidence interval (CI) does not include zero, it indicates a statistically significant indirect effect at the 0.05 level. All regression coefficients reported were (un)standardized. Only those outcome variables revealing statistically significant differences across groups were tested.

## Results

The distribution of sex and age in the intervention group and control group are presented in Table 2.

**Table 2:** The distribution of sex and groups (intervention vs. control) by age.

Age	Participants			
	Boys		Girls	
	Intervention *n* %	Control *n* %	Intervention *n* %	Control *n* %
Overall sample (*n* = 104)	44 64.7	24 35.3	23 63.9	13 36.1
9 years old (*n* = 42)	18 64.3	10 35.7	9 64.3	5 35.7
10 years old (*n* = 17)	7 58.3	5 41.7	5,100	0 00.0
11 years old (*n* = 14)	7 87.5	1 12.5	3 50.0	3 50.0
12 years old (*n* = 31)	12 60.0	8 40.0	6 54.5	5 45.5

A total of 104 participants completed the questionnaire at both pre-test and post-test. Specifically, 67 (64.4%) participants in the intervention group had valid data, and 37 (35.6%) participants had valid data in the control group. A chi-square test of independence was conducted to examine the relationship between sex and group. The results showed no statistically significant association between these variables (χ^2^_1_ = 0.007, *p* = 0.934). Moreover, a chi-square test of independence was conducted to determine the relationship between age and group. The results showed no statistically significant association between these variables (χ^2^_3_ = 1.129, *p* = 0.770).

### Correlations across the included variables

The correlations presented in Table 3 show that there is a strong correlation (*p* < 0.01) between social self-efficacy and social support, as anticipated. This robust relationship aligns with social cognitive theory (e.g., Bandura, 1977, 1986), suggesting that perceived support enhances confidence in social interactions.

**Table 3:** Pearson’s correlations (r) for all included variables (*n* = 104).

Scales	Social Self-efficacy	Social Support	Cog. Appr. Pre	Cog. Appr. Post	Emot. Surp. Pre	Emot. Surp. Post	Perc. Comp. Pre	Perc. Comp. Post	Usef./ value Pre	Usef./ value Post	Int./ enjoy Pre	Int./ enjoy Post
Social Self-efficacy	1											
Social Support	0.609**	1										
Cog. Appr. Pre	−0.271**	−0.249*	1									
Cog. Appr. Post	−0.301**	−0.190	0.502**	1								
Emot. Surp. Pre	0.132	−0.015	−0.167	−0.096	1							
Emot. Surp. Post	0.156	0.233**	0.031	0.085	0.311**	1						
Perc. Comp. Pre	−0.242*	−0.110	0.297**	0.082	−0.058	−0.075	1					
Perc. Comp. Post	−0.246*	−0.064	0.289**	0.319**	−0.177	−0.088	0.546**	1				
Usef./value Pre	−0.088	0.047	0.186	0.043	0.040	0.063	0.492**	0.272**	1			
Usef./value Post	−0.189	0.003	0.169	0.260**	−0.037	0.158	0.252**	0.554**	0.380**	1		
Int./enjoy Pre	−0.164	−0.042	0.255**	0.144	−0.156	−0.024	0.521**	0.358**	0.601**	0.533**	1	
Int./enjoy Post	−0.087	−0.026	0.171	0.265**	−0.114	−0.039	0.317**	0.637**	0.250*	0.649**	426**	1

Social Self-Efficacy = READ Social Self-Efficacy; Social Support = READ Social Support; Cog. Appr. Pre = ERQ-CA Cognitive reappraisal Pretest: Cog. Appr. Post = ERQ-CA Cognitive reappraisal Posttest.*p <.05, **p <.001.

A significant correlation (*p* < 0.01) was also identified between social self-efficacy and emotional reappraisal, measured at both assessment points. This link supports the idea that confident individuals may actively reframe stressors, a key tenet of emotion regulation frameworks.

Furthermore, the results demonstrate a positive and significant correlation between social self-efficacy and perceived competence. This finding indicates that using cognitive reappraisals as a mediator is methodologically appropriate for primary and secondary outcome variables. In contrast, at any assessment point, there were non-statistically significant correlations (*p* > 0.05) between social self-efficacy, cognitive appraisals, and expressive suppression. The negative correlations observed between cognitive appraisals, both at pre- and post-test (Time I and Time II), and expressive suppression at the pre-test, suggest that children who engage in more expressive suppression might rely less on cognitive appraisal. Additionally, the negative, though non-significant, pre-test correlation between cognitive reappraisal and expressive suppression did not persist after the intervention, which may reflect the intervention’s impact on children’s emotion regulation strategies.

### Within-group differences across time

A paired samples t-test was conducted to assess the differences in scores between Time I and Time II for both the intervention and control groups (Table 4). In the intervention group, the results showed that the scores for interest/enjoyment were marginally but significantly lower at Time II compared to Time I (*t* = 2.50, *p* < 0.05). Conversely, in the control group, there was a significant increase in scores for expressive suppression from Time I to Time II (*t* = −2.45, *p* < 0.05), indicating an increased use of this emotional regulation strategy at Time II. There were no statistically significant differences for all other group comparisons.

**Table 4:** Paired sample t-tests (within-groups) and across time (Time I and Time II).

Dependent variables	Intervention group (*n* = 68)				t-test and *p*-values		Control group (*n* = 37)				t-test and *p*-values	
	Time I		Time II		*t*	*p*	Time I		Time II		*t*	*p*
	*M*	*SD*	*M*	*SD*			*M*	*SD*	*M*	*SD*		
Value/Usefulness	6.43	0.55	6.33	0.69	1.10	0.27	6.49	0.54	6.54	0.45	−0.65	0.52
Interest/Enjoyment	6.54	0.48	6.32	0.78	2.50	0.015	6.69	0.38	6.550	0.53	1.46	0.152
Perceived Competence	5.81	0.90	5.77	0.96	0.37	0.71	6.12	0.78	6.19	0.68	−0.68	0.50
Expressive Suppression	10.67	2.32	10.52	3.33	0.37	0.72	9.35	2.84	10.70	2.95	−2.45	0.02
Cognitive Reappraisal	20.72	3.49	21.22	3.76	−1.20	0.23	21.87	3.20	21.95	3.59	−0.13	0.90

*p* < 0.05. Note: S-SE = Social Self-Efficacy; SSF = Social Support Family; CR = Cognitive Reappraisal; ES = Expressive Suppression; PC = Perceived Competence; IE = Interest/Enjoyment; UV = Usefulness Value.

### Examining differences between the intervention and the control groups by applying GLM univariate analysis

An initial general linear model (GLM) univariate regression analysis was carried out to assess the relationship between social self-efficacy (the dependent variable) and categorical predictors, such as groups, sex, and age (Table 5). To control for potential confounding effects, sex, age, and social support were included as covariates. The GLM univariate analysis was selected for its capacity to model fixed effects for categorical predictors and to provide effect sizes (partial eta squared, ηp^2^), with all analyses conducted using SPSS software version 29 (SPSS, Inc., Chicago, IL, USA). The results from the GLM univariate regression indicated that the group significantly influenced social self-efficacy scores (*F*_1,103_ = 6.73, *p* = 0.011, ηp^2^ = 0.064). Interestingly, the control group reported a lower score on the social self-efficacy (primary outcome) variable, indicating an experience of better social self-efficacy. Meanwhile, sex (*F*_1,103_ = 0.24, *p* = 0.628, ηp^2^ = 0.002) and age (*F*_1,103_ = 0.88, *p* = 0.349, ηp^2^ = 0.009) were not significant predictors of social self-efficacy scores. However, social support emerged as a significant predictor (*F*_1,103_ = 60.94, *p* < 0.001, ηp^2^ = 0.381). Including sex, age, and social support as covariates ensured that these demographic variables did not confound the observed differences in social self-efficacy across groups. The overall model explained approximately 41.5% of the variance in social self-efficacy scores (*R*^2^ = 0.415, adjusted *R*^2^ = 0.391). Additionally, when applying perceived competence at post-test, expressive suppression, and cognitive reappraisal as dependent variables, statistically significant differences across groups were identified for perceived competence (indicated by *F*_1,102_ = 5.58, *p* = 0.020, ηp^2^ = 0.054). Notably, none of the three covariates (sex, age, and social support) showed statistical significance in this analysis.

**Table 5:** Means and standard deviations of all dependent variables for the intervention and control conditions and between-group comparisons for the two groups.

Dependent variables	Intervention group (*n* = 68)		Control group (*n* = 36)		Intervention vs. Control group, between-group comparison		
	Mean	SD	Mean	SD	*F-change*	*p*	Eta Squared (η^2^)
Primary outcome measure							
Social self-efficacy	9.147	3.621	7.667	2.651	6.726	0.011*	0.064
Secondary outcome measures							
Perceived competence	5.781	0.957	6.194	0.691	5.583	0.020*	0.054
Cognitive reappraisal	21.201	3.757	22.078	3.605	0.903	0.344	0.037
Expressive suppression	10.567	3.336	10.556	2.853	0.014	0.906	0.000
Control measures	Mean	SD	Mean	SD	*t*	*p*	
Usefulness	6.331	0.693	6.541	0.454	−1.856	0.066	
Enjoyment	6.323	0.772	6.550	0.531	−1.584	0.116	
Social support family	6.279	3.022	6.139	2.045	0.250	0.803	

*indicates statistically significant values with a *p*-value lower than *p* < 0.05 for the primary outcome measure, and *p* < 0.025 for the secondary and control measures.

### Testing for mediation

An assessment of the standardized regression residuals for the dependent variable, self-efficacy, through a normal P–P plot revealed a slight deviation from the normality line. However, the scatterplot displayed no discernible pattern, and the residuals were approximately evenly distributed above and below the vertical and horizontal axes, indicating that the minimum standard for validating the homoscedasticity assumption has been met. In mediation, multicollinearity among predictor variables is commonly expected, although it is acknowledged as a concern specifically in the context of multiple regression (Hayes, 2018).

A mediation analysis was conducted using Hayes’ PROCESS macro (Model 4) to examine whether cognitive reappraisals mediated the relationship between group assignment (intervention vs. control; independent variable) and social self-efficacy (dependent variable), controlling for sex, age, and social support (Table 5). The analysis utilized a sample of 105 participants utilizing bootstrap standard errors from 5,000 random draws to calculate the standard error of the indirect effect. Table 5 displays the relationship model between groups and social self-efficacy as mediated by cognitive reappraisal and expressive suppression. This model explained 42.6% of the total variance (*F*_6, 96_ = 11.874, *p* < 0.001). However, the results indicated no significant indirect effects of the intervention through either mediator.

### Direct and total effects

The intervention showed a significant direct effect on self-efficacy (*b* = −1.31, *SE* = 0.54, *p* = 0.017, 95% CI [−2.39, −0.23]), with the intervention group reporting lower self-efficacy than the control group. The total effect was also significant (*b* = −1.43, *SE* = 0.55, *p* = 0.010, 95% CI [−2.52, −0.35]).

### Indirect effects (mediation pathways)

Neither mediator demonstrated statistically significant indirect effects: cognitive reappraisal: *INDE* = −0.12, boot*SE* = 0.15, 95% CI [−0.49, 0.11] and expressive suppression: *INDE* = −0.001, boot*SE* = 0.06, 95% CI [−0.15, 0.13]. Although cognitive reappraisal accounted for 8.4%, while expressive suppression accounted for only 0.006%, the contrast between mediators was non-significant, *b* = −0.12, *SE* = 0.16, 95% CI [−0.51, 0.16].

### Covariate effects

Covariates did not alter the null mediation findings but ensured that effects were estimated conditional on known predictors. Social support significantly predicted self-efficacy (*b* = 0.77, *p* < 0.001), reducing potential confounding bias in the intervention-outcome relationship. The strong association between social support and self-efficacy suggests that it may operate through unmeasured mechanisms beyond the tested mediators. Furthermore, age and sex were retained as covariates due to their theoretical relevance, despite non-significant effects across all models, thereby maintaining model robustness against omitted variable bias. Table 6 shows the regression results for the mediation model prediction of social self-efficacy.

**Table 6:** Regression results for the mediation model predicting social self-efficacy.

Model	Predictor	b	SE	95% CI	β
Total Effect Model	Group	−1.43*	0.55	[−2.52, −0.35]	−0.43
	Sex	0.31	0.55	[−0.78, 1.40]	0.04
	Age	0.18	0.21	[−0.23, 0.58]	0.07
	Social Support	0.77**	0.10	[0.56, 0.97]	0.59**
Direct Effect Model	Group	−1.31*	0.54	[−2.39, −0.23]	−0.39
	Cognitive Reappraisal	−0.16*	0.07	[−0.30, −0.02]	−0.18
	Expressive Suppression	0.01	0.09	[−0.16, 0.18]	0.01
	Sex	0.30	0.54	[−0.78, 1.38]	0.04
	Age	0.22	0.21	[−0.19, 0.63]	0.09
	Social Support	0.72**	0.11	[0.51, 0.93]	0.55**

Effect type	B	SE	95% Bootstrapped CI
Indirect effects			
Cognitive Reappraisal	−0.12	0.15	[−0.49, 0.11]
Expressive Suppression	−0.001	0.06	[−0.15, 0.13]

*N* = 103. CI = confidence interval; Bootstrapped CI based on 5,000 samples. Sex coded 0 = male, 1 = female. Cognitive Reappr. = Cognitive Reappraisal; Expressive Suppr. = Expressive Suppression. *p < 0.05, **p < 0.001.

## Discussion

This study yielded several notable findings regarding the impact of a targeted intervention on social self-efficacy and related outcomes in children aged 9–12. As hypothesized, post-test analyses revealed a statistically significant difference between the intervention and control groups on the primary outcome measure, social self-efficacy. A statistically significant difference was also observed in one of the secondary measures, perceived competence. In contrast, no statistically significant differences were found for the secondary outcomes of emotional reappraisal and expressive suppression or the primary control variable, social support.

Interestingly, the control group reported higher levels of experienced self-efficacy at post-test compared to the intervention group. While initially counterintuitive, similar findings have been reported in studies also involving younger populations, suggesting that this is not an isolated phenomenon (Adamecz et al., 2025; Kruger and Dunning, 1999; Maj et al., 2024). Several factors may account for this pattern.

First, interventions designed to enhance social self-efficacy and emotion regulation often increase children’s awareness of their strengths and limitations. This heightened self-awareness may lead to more critical self-assessments, resulting in lower self-reported efficacy despite actual skill gains. This aligns with the Dunning-Kruger effect, in which increased competence is accompanied by greater recognition of one’s limitations (Kruger and Dunning, 1999). Limited insight into personal abilities can initially inflate self-perceptions. However, as children learn and reflect, their self-evaluations may become more realistic, sometimes leading to a temporary decline in self-efficacy scores (Pintrich, 1999).

Second, there may be a lag between skill acquisition and confident application in real-world settings (Aune and Stiles, 2009). Although the intervention may have equipped participants with new strategies, they might not yet feel adept at employing these skills outside of the structured environment. Previous research highlights that transferring skills from intervention to daily life requires additional time and support. For example, Wyman et al. (2010) noted that skill generalization is a gradual process, and initial uncertainty is common as children apply new strategies in varied contexts.

Third, learning new emotion regulation strategies may temporarily increase emotional dysregulation. Children experimenting with unfamiliar emotional management approaches may experience short-term setbacks before achieving long-term improvements. This adjustment period can reduce confidence in handling social interactions, as noted by Goldsmith and Kelley (2018) and supported by recent intervention research (Martinsone et al., 2022; Vesely et al., 2022). In addition, interventions that simultaneously address social self-efficacy and emotion regulation may place additional cognitive and emotional demands on participants, potentially leading to stress or feelings of inadequacy during the intervention period. Elevated stress levels have been shown to negatively affect self-efficacy, particularly when individuals adapt to new challenges. Another possible explanation is the comparison effect inherent in group-based interventions. Children may compare their progress to peers who appear to excel, which can foster feelings of inadequacy and lower self-efficacy, especially when peer performance is visible and salient during group activities (Molden and Dweck, 2006).

The pattern of lower self-efficacy in the intervention group was also evident for perceived competence in skill acquisition. Several studies have indicated that the Dunning–Kruger effect phenomenon, in which increased skill or knowledge can temporarily reduce self-perceived competence, also appears in athletic skill acquisition. This effect is observed when individuals, as they gain more training and objective improvement, become more aware of their limitations, leading to a temporary decrease in self-efficacy or perceived competence despite actual progress (Dal et al., 2024; Schescke et al., 2022; Sullivan et al., 2018). Assessing the same sample as in this study, Sørensen et al. (2024) reported that the intervention group demonstrated greater improvement, with a mean improvement of 7.9 s. In contrast, the control group improved by 3.9 s from pre- to post-test. However, subjective measures of competence do not reflect this progress, which supports the notion that increasing self-awareness can temporarily dampen self-perceptions. In accordance with this, investigations have found that athletes’ self-perceptions of competence are influenced not only by their actual abilities, but also by increased self-awareness and meta-perceptions, including how they believe that their coaches and others view their competence (Cecchini et al., 2015). This heightened awareness, especially after targeted interventions or training, can make athletes more critical of their own abilities, mirroring the pattern seen in the Dunning–Kruger effect. Although direct measures of metacognition and calibration were not administered, this interpretation remains plausible and serves as a target for future investigation aimed at refining the mechanisms underlying psychosocial change in youth interventions.

Overall, the observed decreases in social self-efficacy and perceived competence among children receiving the intervention are likely transient and reflect the complex process of skill acquisition, increased self-awareness, and adjustment to new strategies. Similar patterns have been documented in other emotion regulation and self-efficacy interventions (Goldsmith and Kelley, 2018; Pintrich, 1999; Wyman et al., 2010). To advance understanding in this area, future research should prioritize long-term follow-up and explore strategies to facilitate the transfer of learned skills into everyday contexts, as the generalizability of intervention effects remains a key challenge in the field (Ekelund et al., 2023).

Including mediation analyses in evaluating intervention mechanisms is critical for disentangling *how* and *why* specific outcomes emerge. However, both mediators and outcomes were measured contemporaneously at post-test, so mediation results ought to be interpreted as exploratory and do not support strict temporal ordering. Despite this limitation, based on theoretical and earlier reported studies (Ekelund et al., 2023; Prawita and Hariko, 2024), we hypothesized that cognitive reappraisal, a key emotion regulation strategy, would mediate the relationship between intervention participation and improvements in social self-efficacy. While significant correlations were identified between cognitive reappraisal (assessed at baseline and post-intervention) and social self-efficacy (*p* < 0.001), mediation analysis revealed no statistically significant indirect effect of group assignment on social self-efficacy through the use of cognitive reappraisal. This may suggest that the intervention’s total effect on social self-efficacy operates independently of changes in cognitive reappraisal, which is contrary to expectations. The lack of mediation may also reflect a misalignment between the timing of mediator measurement and the intervention’s impact. Cognitive reappraisal requires deliberate practice and meta-cognitive awareness to translate into self-efficacy gains (Cecchini et al., 2015). As noted in prior work, the benefits of emotion regulation strategies often emerge only after participants achieve mastery and integrate these skills into daily interactions. These findings underscore the complexity of intervention mechanisms in youth and highlight the need for nuanced theoretical frameworks when modeling psychosocial outcomes. While cognitive reappraisal did not mediate effects in this study, its significant correlation with social self-efficacy (*p* < 0.001) may suggest that it remains a worthwhile target for refinement in future iterations of the intervention.

Nevertheless, by integrating emotion recognition, breathing strategies, and mindfulness components into football training, the intervention model offers a framework adaptable to various sports and educational settings, facilitating better socio-emotional outcomes through experiential and reflective practice.

### Strengths and limitations of the study

This study is not without certain limitations, but it also possesses several strengths. We applied a randomized controlled trial (RCT) design using a sample of 9 to 12-year-olds employing a specific football technical skill-acquisition program (Sørensen et al., 2024) alongside third-generation low-intensity cognitive behavioral interventions (Aune and Ness, 2025; Pinhas-Hamiel and Hamiel, 2020), capturing the interplay between football skill development and psychosocial processes.

We assessed intervention integrity to ensure competence and adherence to the intervention protocol. Additionally, we employed mediation analysis, which is crucial for understanding the mechanisms underlying intervention effects. Including social support as a covariate was also beneficial in isolating its effect on social self-efficacy while controlling for other variables, thereby improving causal inference and reducing confounders.

Despite using a pre-post design with robust psychometric measurements, we acknowledge that a longitudinal design featuring a skill consolidation phase, multiple post-intervention assessments, and follow-ups could uncover delayed intervention and mediation effects. Further, although the intervention comprised 11 weekly sessions over a four-month period, comparable studies with similar or even shorter durations have documented significant psychosocial effects, especially when accompanied by high-fidelity delivery and targeted psychological components (Gabana et al., 2022; Ng-Knight et al., 2022; Belz et al., 2020). Also, Aelterman et al. (2014) found an effect of a three-month intervention grounded in SDT, and a study of Tessier et al. (2010) found effects on students’ psychological needs (relatedness) after an intervention of only 6 lessons of 1 h. However, like other short-term interventions, sustained change may require longer follow-up or booster sessions. Nevertheless, future studies ought to consider extended or multi-phase interventions.

Our sample design did not include explicit assessment of participants’ previous sporting experience, physical fitness level, or parental motivation, which may act as unmeasured moderators of intervention response. This omission reflects logistical constraints and prioritization of self-efficacy, emotion regulation, and social support as primary covariates. Future research should systematically account for these factors to improve model precision and generalizability.

The absence of baseline measurement for key outcomes prevents pre-existing group differences and weakens causal conclusions, and the findings should be presented as post-intervention associations. Since social support and social self-efficacy were measured only at the post-test, we cannot rule out the possibility of pre-existing differences between the groups in these measures. Nevertheless, minimal differences were observed across other pre-test variables. Furthermore, the social support variable was treated as a control variable, and we did not expect a significant change from pre-test to post-test. As expected, there was no significant difference between the groups on the social support variable at the post-test.

While we incorporated real-world practice during the intervention and encouraged homework assignments, the number of sessions was limited, and we did not assess the frequency or quality of homework application. Planned sessions for parents and guardians were intended to provide them with information on psychological interventions and ways to support their children’s application of learned skills. Unfortunately, these sessions were not realized. Also, low internal consistency in at least one subscale reduces estimate precision and can attenuate true effects of the study. Finally, integrating a self-report assessment for self-criticism could have strengthened the study.

## Conclusion

Self-report measures of self-efficacy (e.g., the Resilience Scale for Adolescents, READ; Hjemdal et al., 2006) and emotion regulation (e.g., the Emotion Regulation Questionnaire for Children, ERQ-CA; Gullone and Taffe, 2012) are widely used, but risk measurement biases. The Dunning-Kruger effect - in which individuals overestimate their abilities (Kruger and Dunning, 1999), may distort self-assessment. Specifically, children in interventions might report lower self-efficacy as they gain awareness of skill gaps, despite objective improvements (Burson et al., 2006). This underscores the need for multi-method assessments (e.g., behavioral observations, peer and guardians’ ratings) to complement self-reports assessing intervention effects among children.

## Data Availability

The raw data supporting the conclusions of this article will be made available by the authors, without undue reservation.
